# Shape and stiffness memory ionogels with programmable pressure-resistance response

**DOI:** 10.1038/s41467-022-29424-z

**Published:** 2022-04-01

**Authors:** Shuyun Zhuo, Cheng Song, Qinfeng Rong, Tianyi Zhao, Mingjie Liu

**Affiliations:** 1 Key Laboratory of Bio-Inspired Smart Interfacial Science and Technology of Ministry of Education, School of Chemistry, Beihang University, 100191 Beijing, People’s Republic of China; 2 School of Chemistry and Phamaceutical Sciences, Guangxi Normal University, 541004 Guilin, People’s Republic of China; 3 Beijing Advanced Innovation Center for Biomedical Engineering, Beihang University, 100191 Beijing, People’s Republic of China; 4 International Research Institute for Multidisciplinary Science, Beihang University, 100191 Beijing, People’s Republic of China; 5 Research institute of frontier science, Beihang University, 100191 Beijing, People’s Republic of China

**Keywords:** Gels and hydrogels, Mechanical properties, Sensors and biosensors

## Abstract

Flexible pressure sensors usually require functional materials with both mechanical compliance and appropriate electrical performance. Most sensors based on materials with limited compressibility can hardly balance between high sensitivity and broad pressure range. Here, we prepare a heterophasic ionogel with shape and stiffness memory for adaptive pressure sensors. By combining the microstructure alignment for stiffness changing and shape memory micro-inclusions for stiffness fixing, the heterophasic ionogels reveal tunable compressibility. This controllable pressure-deformation property of the ionogels results in the pressure sensors’ programmable pressure-resistance behavior with tunable pressure ranges, varied detection limits, and good resolution at high pressure. Broad pressure ranges to 220 and 380 kPa, and tunable detection limit from 120 to 330 and 950 Pa are realized by the stiffness memory ionogel sensors. Adaptive detection is also brought out to monitor tiny pressure changes at low stiffness and distinguish different human motions at high stiffness. Using shape and stiffness memory materials in pressure sensors is a general design to achieve programmable performance for more complex application scenarios.

## Abstract (web summary)

Flexible pressure sensors require functional materials accounting for mechanical compliance and electrical performance simultaneously but sensor materials often suffer from limited compressibility which decreases sensitivity over a large pressure range. Here, the authors demonstrate a heterophase ionogel with shape and stiffness memory for adaptive pressure sensing

## Introduction

Flexible pressure sensors have attracted long-lasting concern due to their promising application in artificial intelligence, wearable medical devices, and soft robots^1–4^. An ideal pressure sensor is usually composed of stable electrodes and functional materials with a combination of mechanical compliance and appropriate electrical performance^1^. The most frequently used matrix materials include conductive polymers and elastomer composites, such as poly(3,4-ethylenedioxythiophene):polystyrene sulfonate (PEDOT:PSS), polyvinylidene difluoride (PVDF), carbon nanomaterials, and polydimethylsiloxane (PDMS)^5–12^. To date, current pressure sensors, including capacitive, piezoelectric, piezoresistive, and triboelectric sensors, have been implemented with well-designed or micro-structured flexible matrix materials for high performance^13–19^. However, most pressure sensors suffered from a relatively narrow pressure range due to the matrix materials’ inherent limitation of compressibility. Especially under high pressure, such sensors with low sensitivity usually lost sensing ability in a highly compressed state, which severely restricted their further applications. Recent works have provided insights on improving the compatibility of sensitivity and pressure range by using phase transition composites or graded architectures as the active matrix materials with controllable mechanics and compressibility^20,21^. Materials with tunable stiffness have an adaptive pressure-deformation relationship, which may lead to different pressure-resistance performance of the pressure sensors^22,23^. Therefore, developing adaptive materials with tunable stiffness is a promising solution for pressure sensors to balance between pressure range and sensitivity, and to enhance the intrinsic sensor performance.

Here, we reported a shape and stiffness memory ionogel for adaptive pressure sensors with programmable pressure response. The heterophasic ionogels consisted of conductive ionogel frameworks containing ionic liquids (ILs) and uniformly dispersed poly(stearyl methacrylate) (PSMA) micro-inclusions. The isotropic microstructure of the ionogels could change to anisotropic microstructure by compressing, which was then fixed thanks to the shape memory effect of the phase transition micro-inclusions. The synergistic design of combining the microstructure alignment for stiffness changing and shape memory micro-inclusions for stiffness fixing, led to the shape and stiffness memory of the heterophasic ionogels. It is worth noting that such shape and stiffness memory property was rarely studied in conventional shape memory polymers. Along with stiffness memory/recovery, the compressibility (pressure-deformation behavior) of the ionogels varied. As a result, the programmable pressure-resistance response was realized by the ionogel-based sensors with tunable pressure range, detection limit, and resolution. The pressure range broadened from 60 to 200 and 380 kPa as the stiffness of the ionogel sensors increased. The ionogel sensors also showed an increased detection limit (from 120 to 330 and 950 Pa) and good resolution at high stiffness. Besides, the unique physicochemical properties of ILs endowed the heterophasic ionogels anti-freezing property and conductivity in a broad temperature range (−80 to 60 °C). The ionogel pressure sensors had high sensitivity, an ultrafast response time of 9.43 ms, and anti-fatigue performance. Based on the stiffness memory of the heterophasic ionogels, our ionogel pressure sensors could detect subtle details of human physiological signals at low stiffness, and have good pressure resolution at high stiffness. Adaptive detection was also brought out to distinguish different human motions (stepping, walking, and jumping), which indicated a promising application in medical equipment and soft robots.

## Results

### Heterophasic ionogels with shape and stiffness memory

In living tissues, cellular and extracellular matrix stiffness is critical for diverse physiological and pathological processes, such as cell migration and vascular disease^24,25^. It was found that endothelial cells could memorize the high stiffness caused by high glucose. Such a “stiffness memory” effect led to a stable mechanical alteration of the endothelial monolayer without being influenced by the changing environment. Inspired by this phenomenon, artificial materials with stiffness memory could possess high adaptability to tune mechanical property on-demand in practical applications.

**Fig. 1:**
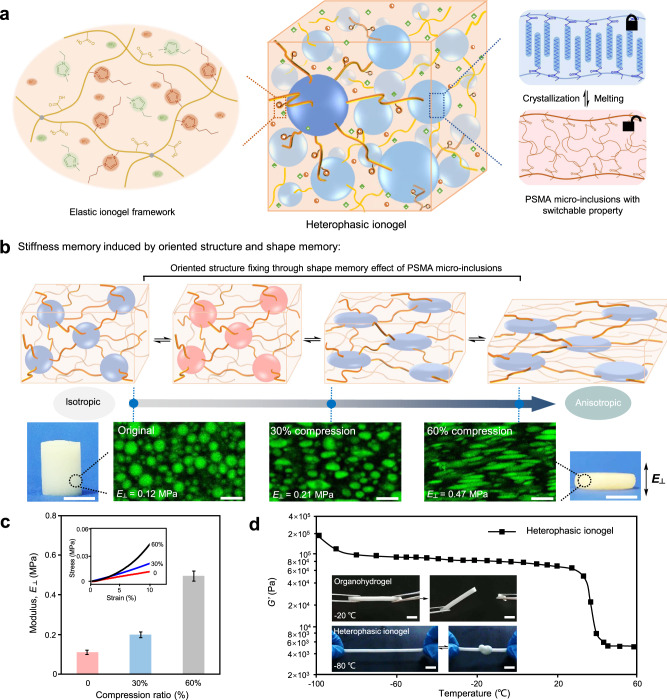
Schematic illustration and stiffness memory of the heterophasic ionogels. **a** The heterophasic ionogels composed of elastic ionogel frameworks and switchable poly(stearyl methacrylate) (PSMA) micro-inclusions. **b** Stiffness memory effect of the ionogels by fixing the oriented structure utilizing the shape memory effect of the PSMA micro-inclusions. Pictures showing the original and compressed ionogel samples, scale bar, 1 cm. The CLSM images indicated the structure change from isotropic to anisotropic of the heterophasic ionogels with different modulus, *E*_*┴*_ (modulus perpendicular to the orientation direction), scale bar, 10 μm. **c** Mechanical properties of heterophasic ionogels at different compression ratios. The enlarged figure clearly showed the enhanced stress of the compressed samples. The error bars represent 1 SD (*n* = 3). **d** The storage moduli (*G’*) of the heterophasic ionogels in a wide temperature range. Inset pictures revealed the anti-freezing behavior of the heterophasic ionogel and the organohydrogel, scale bar, 1 cm.

To achieve such materials with stiffness memory, components with reversible stimuli-response play an important role in memorizing the programmed stiffness^26–28^. Here, we choose a simple phase change component, PSMA, as the responsive unit to constructed adaptive materials with stiffness memory. As shown in Fig. 1a, the heterophasic ionogels contained the elastic ionogel frameworks and the switchable PSMA micro-inclusions, which were fabricated through in-situ UV polymerization of a stable oil/IL emulsion. The ionogel framework was polymerized from acrylic acid (AA) and 1-butyl-3-vinylimidazolium tetrafluoroborate (VBIMBF_4_), with 1-ethyl-3-methylimidazolium tetrafluoroborate (EMIMBF_4_) and ethylene glycol as the solvents; and the PSMA micro-inclusions featured reversible melting/crystallization transition. The microstructure of the ionogels, visualized using confocal laser scanning microscopy (CLSM), revealed a stable heterophasic network and uniform dispersion of the PSMA micro-inclusions with the size of 3–5 μm (Supplementary Fig. 1). Previous works have proved that the phase transition PSMA micro-inclusions could endow the ionogels shape memory effect^26^. Differential scanning calorimetry (DSC) curves in Supplementary Fig. 2 demonstrated sharp peaks around 35 °C, which can be ascribed to the crystallization/melting phase transition of PSMA micro-inclusions. As a result, the heterophasic ionogels showed good shape memory performance with high shape fixity (*R*_*f*_) and shape memory (*R*_*r*_).

In materials processing field, oriented structures with extended chains were obtained at high deformation ratio to achieve enhanced stiffness and strength of the processed samples^29^. For rigid shape memory polymers, obvious stiffness change was hard to get at small deformations due to the frozen polymer conformation and intrinsic high stiffness at glassy or crystalline state. Soft materials with low modulus were more susceptible to elastic deformations because of entropic elasticity. Herein, we combined the stiffness changing of the microstructure alignment and stiffness fixing of the shape memory units together in our ionogels. We programmed the microstructure of the heterophasic ionogels by simply compressing at 45 °C (higher than the melting temperature, *T*_m_, of PSMA) and fixing at 10 °C (lower than the crystallization temperature of PSMA). As shown in Fig. 1b, the original ionogels revealed isotropic microstructure with uniform dispersion of the micro-inclusions, while the compressed ionogels showed anisotropic microstructure perpendicular to the direction of compression. The changes of the microstructure were observed from the CLSM images. The spherical PSMA micro-inclusions turned to be oblate with highly oriented dispersion after compression. In addition, the alignment of the ionogel network was also observed by the polarizing optical microscope (POM) with strong interference color shown in compressed samples and dark in original samples (Supplementary Fig. 3). The anisotropic nature of the compressed ionogels led to higher modulus (*E*_┴_) perpendicular to the orientation due to entropic elasticity, as demonstrated in Fig. 1c. As a result, the *E*_┴_ increased from 0.12 to 0.21 and 0.47 MPa when the original ionogels compressed by 30% and 60%, respectively. Based on the high shape fixity of the heterophasic ionogels, the programmed anisotropic microstructure and varied stiffness were easily memorized at low temperature. Therefore, combining the oriented microstructure-induced stiffness variation and shape memory effect, the heterophasic ionogels showed stiffness memory property.

### Switchable mechanics and anti-freezing property

In addition to endowing the heterophasic ionogels shape memory property, the rigid PSMA micro-inclusions, acting as cross-linkers, enhanced the ionogels’ mechanical performance at the crystalline state, as demonstrated in Supplementary Fig. 4. The phase transition behavior of the PSMA micro-inclusions from rigid crystals to elastic quasi-static matter led to the switching mechanics of the heterophasic ionogels. Both rheological and tensile tests displayed a drop in modulus or strength as temperature raised above *T*_m_ (Fig. 1d and Supplementary Fig. 5). For example, the modulus of the original heterophasic ionogels decreased from 8 × 10^4 ^Pa at 20 °C to 4 × 10^3 ^Pa at 60 °C. Supplementary Fig. 6 also showed the reversibly switched mechanics between a rigid state and a flexible, stretchable state.

Freezing and volatilization of the solvent in gels are the main challenges that severely limit their practical applications. In our work, we chose low-freezing point ILs as the anti-freezing components. The glass transition temperature of poly(AA-VBIMBF_4_) and EMIMBF_4_ were measured to be −79 and −102 °C, respectively, resulting in good anti-freezing property of the ionogels with stable mechanics and elasticity at subzero temperatures^30^. As shown in Fig. 1d, the original heterophasic ionogel had high flexibility to be stretched and knotted at −60 °C, and its storage modulus remained stable at temperatures from 25 to −80 °C. By contrast, the organohydrogels with hydrogel framework became fragile and broken to pieces at −20 °C, due to water freezing at subzero temperatures. The heterophasic ionogels also showed high stability with no obvious change or weight loss after exposure in the air for 30 days, because of the low vapor pressure of the ILs and the stable heterophasic structure, as shown in Supplementary Fig. 7. These heterophasic ionogels possessed shape memory effect from the PSMA micro-inclusions and unique properties from the ILs, such as anti-freezing capability, high conductivity, and high stability.

### Sensing performance of the ionogel pressure sensors

**Fig. 2:**
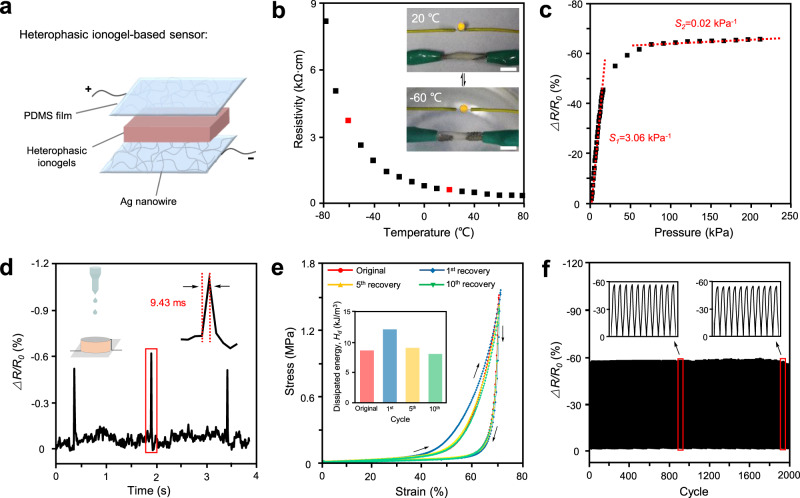
The pressure-resistance response of the heterophasic ionogel sensors. **a** Components of the heterophasic ionogel pressure sensors. **b** Resistivity of the heterophasic ionogels in the temperature range from −80 to 80 °C. The pictures showed that the heterophasic ionogels could work as wires at −60 °C. Scale bar, 1 cm. **c** Relative resistance variation (*∆R*/*R*_*0*_) and sensitivity (*S*) of the heterophasic ionogel sensors under different pressures. **d** The dynamic response time of the heterophasic ionogel sensor to the fallen droplet. **e** First loading-unloading cycle of original and recovered heterophasic ionogels at 20 °C. Inset showed the dissipated energy of these samples. **f** Cyclic tests showing the stable response of the heterophasic ionogels.

In addition to controllable mechanics, sufficient flexibility, and stable conductibility in a wide temperature range, it is also necessary for pressure sensors to have high sensibility, fast response time, and an adequate pressure-responsive range. As illustrated in Fig. 2a, we fabricated pressure sensors using PDMS film with Ag nanowire layer, the original heterophasic ionogels (*E*_┴_ = 0.12 MPa), and conductive wire. The good anti-freezing property and high conductivity of the IL components ensured the possibility of the ionogel sensors to work in a wide temperature range even at −60 °C (Fig. 2b). To measure the sensing performance of the ionogel pressure sensors, relative resistance change (*∆R*/*R*_*0*_) was investigated in a broad pressure range. From Fig. 2c, the sensibility of the heterophasic ionogel-based sensor was calculated to be 3.06 kPa^−1^ in the pressure range of 0–60 kPa. However, it dropped sharply to 0.02 kPa^−1^ at high pressures of 60–200 kPa due to the limited deformability of the ionogels. Notably, the as-prepared pressure sensors exhibited high linearity with *R*² = 0.9929 and a negligible hysteresis phenomenon owning to the elastic ionogel framework, which indicated that the heterophasic ionogels were suitable for constructing a pressure sensor (Supplementary Figs. 8 and 9). Such ionogel pressure sensors could be used in detecting external stimuli, such as a small drop of water. The amplified signal in Fig. 2d revealed the ultrafast response time of 9.43 ms, which was preferred by precision devices, wearable sensors, and artificial intelligence.

Based on the high sensibility and ultrafast response time, the heterophasic ionogel pressure sensors revealed a high potential in monitoring human motions and physiological conditions. As shown in Supplementary Fig. 10, the original ionogel-based pressure sensors showed stable performance in detecting the wrist pulse pressure, walking, and bending a finger. In specific, when the human subject turned from calm to excited, the output signal changed obviously from 16% to 28% due to the higher pulse pressure at the excited state. Notably, the amplified signal of the wrist pulse clearly showed the typical peak characteristics, that is, percussion wave (P_1_), tidal wave (P_2_), and valley-diastolic wave (P_3_), which linked with blood pressure and heart rate^31,32^. Therefore, our pressure sensors may help us observe and estimate a person’s physiological state for clinical diagnosis and disease prevention.

### Anti-fatigue and self-recovery performance

To meet the needs for high adaptability of electronic devices, the conductive materials were expected to have anti-fatigue and self-recovery capabilities. Loading-unloading tests conducted on the original ionogels showed the areas of the hysteresis loops, which increased with larger compressive strain and decreased sharply from 8.50 kJ/m^3^ (1st cycle) to 2.36 kJ/m^3^ (2nd cycle) under 70% compression (Supplementary Figs. 11 and 12). This could be ascribed to the rigid PSMA micro-inclusions with lack of sufficient energy storage/dissipation mechanism and possible micro-cracks inside under high compression ratios^33^. During the 2nd to 10th loading-unloading cycles, the areas of the hysteresis loops were almost constant, demonstrating that the heterophasic ionogels did not undergo more damage and exhibited fatigue resistance.

Notably, the heterophasic ionogels displayed self-recoverable mechanics by thermal stimuli. After the heterophasic ionogel was heated to 45 °C and then cooled at 5 °C, the hysteresis loops recovered to the original state. The dissipated energies of the self-recovered ionogels were calculated to be 12.05, 9.07, and 8.03 kJ/m^3^ after 1st, 5th and 10th recovery, which indicated good mechanical recovery ability, as shown in Fig. 2e and Supplementary Fig. 13. Accelerated entanglement of polymer chains at high temperatures and strong hydrophobic interactions of PSMA promoted the recovery process. The ionogel sensors also showed a stable pressure-resistance response during 2000 cycles under various pressures, as shown in Fig. 2f and Supplementary Fig. 14, showing good reproducibility and anti-fatigue of the pressure sensors.

### Wide pressure range and tunable detection limit

**Fig. 3:**
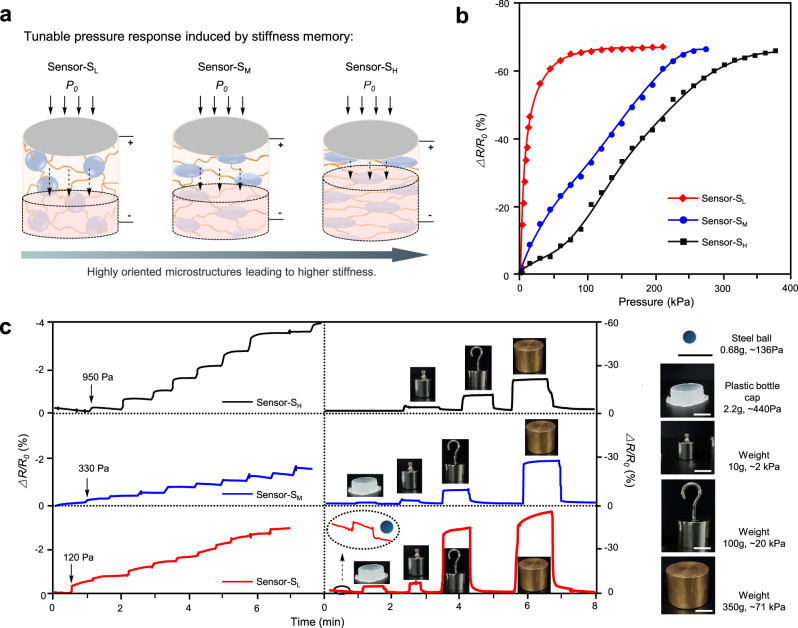
Wide pressure range and tunable detection limit of the ionogel pressure sensors. **a** Illustration showed the pressure-response change with varied stiffness of the heterophasic ionogels. Sensors with oriented structure and higher stiffness revealed less deformation under the same pressure. **b** Pressure-resistance response of the sensors with low (sensor-S_L_), middle (sensor-S_M_), and high stiffness (sensor-S_H_) in the pressure range from 0 to 400 kPa. **c** Detection limit of the pressure sensors with different stiffness. The three sensors responded differently to a series of pressure objects: steel ball, plastic bottle cap, and weights. Scale bar, 1 cm.

With the rapid development and application of pressure sensors, more and more concerns have paid to broadening the pressure-response range and improving adaptability. To date, shape memory polymers and phase change materials are utilized in flexible electronics and sensors to achieve tunable property and wide application scope^22,23,34–36^. Owing to the stiffness memory property of the heterophasic ionogels, the pressure sensors were expected to possess tunable pressure-response performance. To test this assumption, we constructed three ionogel pressure sensors with different stiffness, that were sensor-S_L_ (*E*_┴_ = 0.12 MPa), sensor-S_M_ (*E*_┴_ = 0.21 MPa), and sensor-S_H_ (*E*_┴_ = 0.47 MPa). As shown in Supplementary Fig. 15, the *∆R*/*R*_*0*_ value of sensor-S_L_ increased quickly as pressure increasing to 43 kPa, and then remained stable at higher pressures. While sensor-S_M_ and sensor-S_H_ responded differently to increasing pressures in the whole pressure range (8–64 kPa). It was because that the sensors with higher stiffness remained compressible with smaller resistance change at high pressure (Fig. 3a). To further investigate the sensing mechanism, pressure responses of sensor-S_L_, sensor-S_M_, and sensor-S_H_ were studied in a wide pressure range from 0 to 400 kPa. From Fig. 3b, sensor-S_L_ almost lost detecting capability due to the low sensibility of 0.02 kPa^−1^ at pressure >60 kPa. The pressure ranges of sensor-S_M_ and sensor-S_H_ broadened to 220 kPa and 380 kPa with the sensibility of 0.28 and 0.12 kPa^−1^, respectively. Stiffer sensors could withstand higher pressure till fully compressed. The wide pressure range would greatly increase detection capability and broaden the application scope of the ionogel pressure sensors.

The detection limit is also an important parameter to evaluate the capability of pressure sensors for a certain application. Interestingly, we found that the detection limit varied with the stiffness of the pressure sensors. As shown in Fig. 3c, the detection limits were tested to be 120, 330, and 950 Pa for sensor-S_L_, sensor-S_M_, and sensor-S_H_, respectively. Large pressure was required by stiffer sensors to produce sufficient deformation for the minimum resistance change. As a result, pressure sensors could shield or detect pressures on demand. For example, sensor- S_L_ could detect light items, like a small steel ball (136 Pa) and a plastic bottle cap (440 Pa), while sensor-S_H_ only respond to heavy items. Therefore, we can tune the detection limit of the ionogel pressure sensors through stiffness memory to adapt to a given working environment.

### Adaptive detection performance

**Fig. 4:**
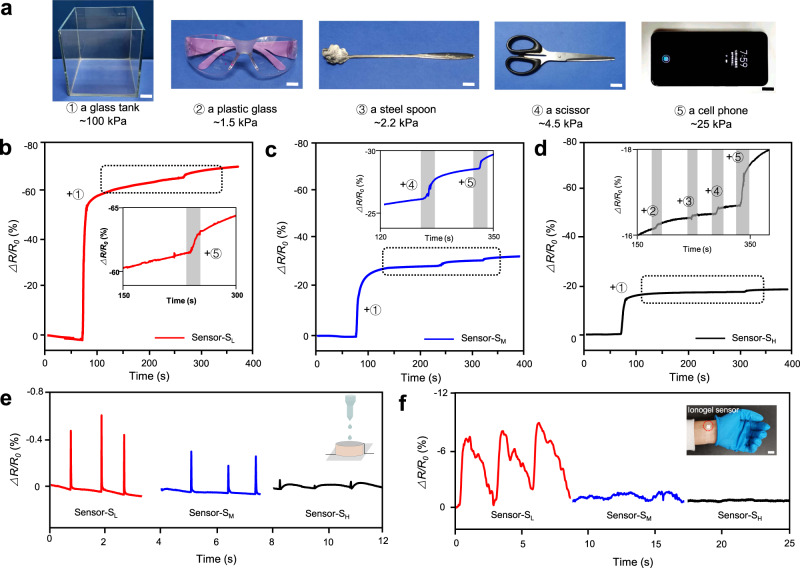
Pressure resolution of the ionogel sensors with different stiffness at high pressures. **a** Pressure objects: a glass tank of 100 kPa, a plastic glass of 1.5 kPa, a steel spoon of 2.2 kPa, a scissor of 4.5 kPa, and a cell phone of 25 kPa. Pressure responses of **b** sensor-S_L_, **c** sensor-S_M_ and **d** sensor-S_H_ to different pressure objects under a base pressure of 100 kPa. The three sensors could record some or all of the loaded objects. Detection of tiny pressure changes of **e** falling droplet and **f** human pulse by sensor-S_L_, sensor-S_M_, and sensor-S_H_. Scale bar, 1 cm.

Many pressure sensors tended to lose sensing ability as the resolution decreasing due to limited compressibility of the matrix materials at high pressures. Sensors with high resolution at high pressures are expected to expand the application scope. To investigate the relationship between resolution and stiffness of ionogel sensors, a series of experiments were carried out on sensor-S_L_, sensor-S_M_, and sensor-S_H_ (Fig. 4a). Firstly, we put a glass tank (~100 kPa) on the pressure sensor, then we gently put a plastic glass (~1.5 kPa), a steel spoon (~2.2 kPa), a scissor (~4.5 kPa), and a cell phone (~25 kPa) in sequence into the glass tank. The corresponding resistance change was shown in Fig. 4b, c, d. At a reference pressure of 100 kPa, sensor-S_L_ and sensor-S_M_ could not detect the small loads of 1.5 and 2.2 kPa, while sensor-S_H_ with high stiffness successfully recorded all pressure changes induced by the plastic glass, steel spoon, scissor, and cell phone. The ionogel pressure sensors with high stiffness were more suitable to work at high pressures due to better pressure resolution and broad pressure range. At low pressures, sensors with small stiffness possessed a lower detection limit and higher sensibility, indicating that these sensors were more qualified in low-pressure applications. As shown in Fig. 4e, f, sensor-S_L_ displayed more details of tiny pressure changes when monitoring human pulse and water drop. These tunable pressure responses, including pressure range, detection limit, and sensibility, greatly enhanced the adaptability of the ionogel pressure sensors for complex applications requiring adjustable sensing capability.

**Fig. 5:**
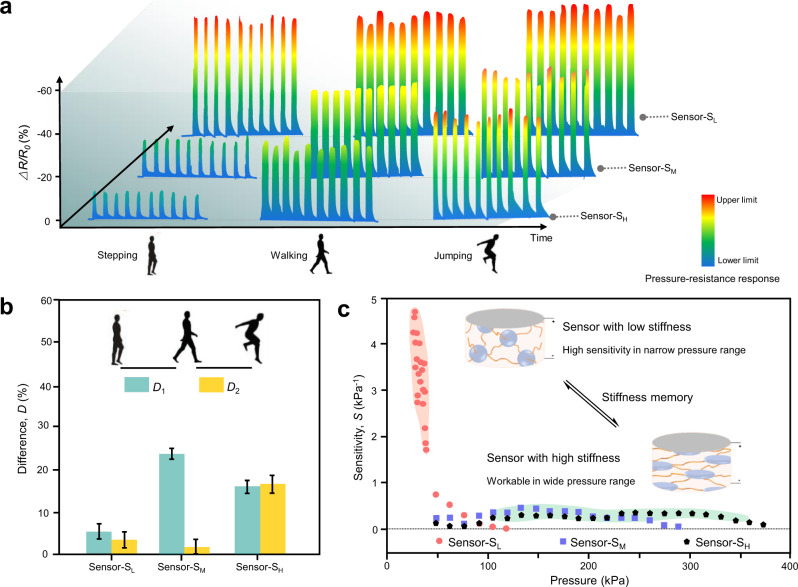
Adaptive detection performance of the heterophasic ionogel sensors through stiffness memory. **a** Resistance signals of the ionogel sensors in detecting different human motions: stepping, walking, and jumping. Scale bar reflected the detection limit of the sensors and ∆*R*/*R*_*0*_ value under different human motions. The upper limit (red) indicated that sensors lost detecting capability with increasing pressure. **b** Difference of resistance change (*D*) showing the capability of the sensors to distinguish the three motions of stepping, walking, and jumping. The error bars represent 1 SD (*n* = 10). **c** Adaptive sensing could be realized with high sensitivity and wide pressure range through stiffness memory of the heterophasic ionogel pressure sensors.

Based on the tunable pressure-resistance relationship, we used these adaptive ionogel sensors to monitor, distinguish, and diagnose the state of the target. As shown in Fig. 5a, three human motions, including stepping, walking, and jumping, were detected using sensor-S_L_, sensor-S_M_, and sensor-S_H_. It showed that the pressure sensors with different stiffness responded differently to various human motions. The ∆*R*/*R*_*0*_ values of sensor-S_L_ caused by stepping, walking, and jumping were −50.76 ± 1.25%, −55.50 ± 2.19%, and −58.80 ± 1.66%, respectively, which were close to the upper detecting limitation, making it hard to distinguish the three motions from each other. While sensor-S_M_ and sensor-S_H_ with wide pressure range (0–220 kPa and 0–380 kPa) realized partial and total distinguish of the three motions, with the output signal of −17.71 ± 0.94%, −42.18 ± 1.29%, −43.73 ± 2.37%, and −12.32 ± 0.77%, −28.92 ± 1.93%, −44.87 ± 1.79%, respectively. The difference (*D*) in the resistance signal of the three motions was calculated in Fig. 5b, where *D*_1_ = (∆*R/R*_*0*_)_stepping _− (∆*R/R*_*0*_)_walking_, and *D*_2_ = (∆*R/R*_*0*_)_walking_ − (∆*R/R*_*0*_)_jumping_. Pressure sensor-S_H_ with large *D* values between stepping, walking, and jumping was suitable to tell apart various stimuli in a wide pressure range. The sensors with tunable stiffness showed adaptive resistance-pressure responses corresponding to varied application environments. The sensing properties could be reversibly switched between high sensitivity and wide pressure range (0–380 kPa) through stiffness memory effect of the heterophasic ionogels, as illustrated in Fig. 5c. Such adaptive pressure sensors based on the heterophasic ionogels will be a promising candidate for future applications in soft robots, wearable devices, and artificial intelligence.

## Discussion

To conclude, we prepared the shape and stiffness memory heterophasic ionogels for adaptive pressure sensors with programmable pressure-resistance response. Combining the oriented microstructure-induced stiffness variation and shape memory effect of the PSMA micro-inclusions, the resultant heterophasic ionogels possessed both shape and stiffness memory effect. Based on the tunable stiffness and compressibility, the heterophasic ionogels had different pressure-deformation responses. As a result, the ionogel-based pressure sensors exhibited programmable pressure-resistance response through manipulating the microstructure of the ionogels by compressing and fixing. Tunable pressure-response ranges up to 380 kPa, adaptive detection limit from 120 to 950 kPa, and good resolution were achieved by the heterophasic ionogel sensors through stiffness memory. Such ionogel pressure sensors could realize adaptive detection in recording tiny pressure changes of human physiological signals at low stiffness or distinguishing different human motions (stepping, walking, and jumping) at high stiffness. The ionogel pressure sensors also showed high sensibility, an ultrafast response time of 9.43 ms, and anti-freezing property. In this paper, we proposed a synergistic design to fabricate shape and stiffness memory ionogels and constructed adaptive pressure sensors with programmable pressure response for applications in varying environments.

## Methods

### Materials

Stearyl methacrylate (SMA), ethyleneglycol dimethacrylate (EGDMA), acrylic acid (AA), 2,2′-diethoxyacetophenone (DEOP), 1-ethyl-3-methylimidazolium tetrafluoroborate (EMIMBF_4_), 1-butyl-3-vinylimidazolium tetrafluoroborate (VBIMBF_4_), and glycol were purchased from Sigma-Aldrich. Poly (ethylene glycol)-block-poly (propylene glycol)-block-poly (ethylene glycol) (PEG-PPG-PEG, average Mn~12600, as an emulsifier) was purchased from Aladdin. CO. N,N′-Bis (2,6-diisopropylphenyl)-1,6,7,12-tetraphenoxy-3,4,9,10-perylenetetracarboxylic diimide (Perylene Red) was purchased from Tokyo Chemical Industry (TCI). Silver nanowire (40 nm in diameter, 2-20 μm in length) was purchased from Jining Leadernano Tech. (L.L.C.). Polydimethylsiloxane (PDMS, Sylgard 184) was purchased from Dow Corning. All the chemicals were used as purchased without further purification.

### Synthesis of the heterophasic ionogels

The heterophasic ionogels were prepared by in-situ polymerization of an emulsion. Firstly, 0.5 g EMIMBF_4_, 0.1 g glycol, 0.1 g AA, 0.3 g VBIMBF_4_, 0.1 mg of EGDMA, 1 μL of DEOP and 0.1 mg of PEG-PPG-PEG were mixed together to form an IL phase. An oil phase, containing 1.2 g of SMA monomer, 3% molar ratio of SMA crosslinker EGDMA, and 5 mg of DEOP, was added to the IL phase at 30 °C and homogenized at a speed of 18,000 rpm for 5 min to form a stable oil-in-IL emulsion. Finally, heterophasic ionogels were synthesized from this emulsion by in-situ photopolymerization at 40 °C for 1.5 h under the protection of N_2_.

#### Synthesis of IL-gels

0.5 g EMIMBF_4_, 0.1 g glycol, 0.1 g AA, 0.3 g VBIMBF_4_, 0.1 mg of EGDMA, 1 μL of DEOP were mixed together. The IL-gel was obtained from this mixture after polymerized under UV irradiation for 1.5 h.

#### Synthesis of organohydrogel

The organohydrogel was fabricated through replacing EMIMBF_4_ and glycol with water. The aqueous phase includes 0.6 g deionized water, 0.1 g AA, 0.3 g VBIMBF_4_, 0.1 mg of EGDMA, 1 μL of DEOP and 0.1 mg of PEG-PPG-PEG. The oil phase and preparation were the same with that of the heterophasic ionogels.

### Fabrication of the pressure sensors

The heterophasic ionogel-based pressure sensor was composed of PDMS film with Ag nanowire layer as the electrodes, the heterophasic ionogels, and silver wire. Two pieces of PDMS films attached on the surfaces of the ionogel, then the silver wires were fixed between the surfaces of PDMS film and the heterophasic ionogels. The PDMS films were firmly fixed on the ionogel after heating to 40 °C due to the viscidity of the ionogel at high temperatures. The sensor size was 2 mm Height × 7 mm Length × 7 mm Width for detection limit, resolution tests, and human pulse test. Besides, the sensor size was 2 mm in height and 3 mm in radius for all the other tests.

## Supplementary information

Supplementary Information

## Data Availability

The data generated in this study are provided in the article and Supplementary Information.
