# Supplementary material for: Shape and stiffness memory ionogels with programmable pressure-resistance response
Source: Nat Commun. 2022 Apr 1;13:1743. doi: 10.1038/s41467-022-29424-z (PMC8976034; doi:10.1038/s41467-022-29424-z)
Supplement: Supplementary file 1 — Supplementary Information [file 41467_2022_29424_MOESM1_ESM.pdf]

Supplementary Information for

Shape and stiffness memory ionogels with programmable pressure-resistance response

Shuyun Zhuo[1], Cheng Song[1], Qinfeng Rong[2], Tianyi Zhao[1*] and Mingjie Liu[1,3,4,5*]

*Corresponding author. Email: zhaoty@buaa.edu.cn, liumj@buaa.edu.cn

This PDF file includes:

Supplementary Note 1. Characterization of microstructure.

Supplementary Note 2. Differential scanning calorimeter (DSC) measurements.

Supplementary Note 3. Measurements of rheological properties.

Supplementary Note 4. Measurements of shape memory properties.

Supplementary Note 5. Measurements of mechanical properties.

Supplementary Note 6. Measurements of self-recovery capacity.

Supplementary Note 7. Measurements of pressure-resistance performance of the ionogel pressure sensor.

Supplementary Figure 1. Structure characterization of the heterophasic ionogels.

Supplementary Figure 2. Shape memory property of the heterophasic ionogels.

Supplementary Figure 3. POM images of the ionogel framework.

Supplementary Figure 4 Strain-stress curves of the heterophasic ionogel and ionogel framework.

Supplementary Figure 5. Strain-stress curves of the heterophasic ionogels.

Supplementary Figure 6. Switchable mechanics of the heterophasic ionogels.

Supplementary Figure 7. Weight and structure changes of the heterophasic ionogels.

Supplementary Figure 8. Resistance change of the ionogel sensors.

Supplementary Figure 9. Resistance changes of the ionogel sensors under different strain and pressure.

Supplementary Figure 10. Resistance changes of the ionogel sensors in monitoring physiological signals.

Supplementary Figure 11. The cyclic loading-unloading compressive tests of the heterophasic ionogels.

Supplementary Figure 12. The cyclic loading-unloading compressive tests of original ionic gels.

Supplementary Figure 13. The cyclic loading-unloading compressive tests of recovered ionogels.

Supplementary Figure 14. The durable strain-sensitive test.

Supplementary Figure 15. Resistance response of the ionogel sensors under various pressure.

Supplementary Note 1. Characterization of microstructure

The oil-in-IL emulsion was characterized using an optical microscope (Nikon LV100N). Polarized optical microscope (Leica DC 300) was used to observe the isotropic and anisotropic structure of the ionogels. The microstructures of the heterophasic ionogel samples were characterized using a scanning electron microscopy (SEM, JEOL-7500 apparatus, at an accelerating voltage of 5 kV) and a confocal laser scanning microscopy (CLSM, Olympus FV1000-IX81 confocal laser biological microscope). For CLSM measurements, the IL-gels were stained with the hydrophilic dyes (DAPI, blue), and the microrganogel domains were stained with the oleophilic dyes (Perylene Red, red).

Supplementary Note 2. Differential scanning calorimeter (DSC) measurements

DSC measurements were conducted on the PerkinElmer Pyris 1 TGA under nitrogen atmosphere. The original heterophasic ionogel samples, P(AA-VBIMBF$_4$), and EMIMBF$_4$, sealed in aluminum pans, were scanned between -150 and 100°C at a scanning rate of 10 °C/min.

Supplementary Note 3. Measurements of rheological properties

The rheological properties of the original heterophasic ionogel samples were investigated on an Anton Paar model MCR-301 rheometer. The heterophasic ionogel samples in form of sheets (15 mm diameter × 2 mm height) were set under a 15 mm diameter parallel plate. Under a variety of temperatures, the storage moduli (G') of the heterophasic ionogels were swept at 15.8 rad s^{-1} and a constant strain (γ) of 0.1 %. In cyclic tests, the storage moduli were repeatedly measured through heating-cooling the original ionogel samples (0 and 50 °C).

Supplementary Note 4. Measurements of shape memory properties

Compressing/recovering test was conducted on cylinder-shaped (6 mm diameter × 5 mm height) ionogel samples to evaluate shape memory effect. The samples were compressed by 70%

at 45 °C and cooled at 5 °C for 5 min with compression force on. Then the external force was removed to record the height of the fixed samples. With temperature increasing and remaining 45 °C for 5min, the samples recovered. Shape fixity (R_f) and shape recovery (R_r) were calculated to indicate the shape memory property. $R_f = 100\% \times (h_0\text{-}h_f)/(70\% h_0)$, $R_r = 100\% \times (h_r\text{-}h_f)/(h_0\text{-}h_f)$, where h_0, h_f, h_r represent the original height, fixed height, and recovered height.

Supplementary Note 5. Measurements of mechanical properties

The compression and tensile measurements of heterophasic ionogels at varying temperatures were performed using a tensile-compressive tester with a 1000 N load cell (Tensilon HZ-1004D, Hengzhun Instrument Technology Co., China). In tensile test, cylinder-shaped (6 mm diameter × 30 mm height) original samples were stretched at a strain rate of 10 mm min^{-1} up to a maximum strain ε_m. In compression tests, cylinder-shaped (6 mm diameter × 5 mm height) original and compressed samples were measured at a constant compression rate of 10 mm min^{-1}. The modulus was calculated from the linear slope of the stress-strain curves between 0-10 % compression.

Supplementary Note 6. Measurements of self-recovery capacity

Cyclic loading-unloading test was carried out to evaluated self-recoverable property of the original heterophasic ionogels. The cylinder-shaped (6 mm diameter × 5 mm height) original samples were compressed by 70% at a constant loading/unloading rate of 10 mm min^{-1}. Then these samples were heated to 45 °C for 2 hours and cooled at 5 °C for 2 hours. At last, the self-recovered samples were tested again to show the recovery capability of the heterophasic ionogels.

Supplementary Note 7. Measurements of pressure-resistance performance of the ionogel pressure sensor

Resistance changes was recorded by a HIOKI IM3536 LCR meter. The prepared pressure sensors were precisely compressed and released in a controlled manner using a tensile-

compressive tester with a 100 N load cell (Tensilon HZ-1004D, Hengzhun Instrument Technology Co., China) and a computer-controlled force gauge (ESM301 from Mark-10 Corporation). All of the pressure-resistance tests were conducted in an ambient environment, and the compression rate was set at 20 mm min^{-1}. In the human motions test, the sensors were fixed under the shoe sole. To test the detection limit of the pressure sensors, we use a computer-controlled force gauge to apply a stepped pressure of 0.01 N to the sensors until obvious and repeatable resistance change appeared. To ensure the accuracy, every test was repeated three times using different samples.

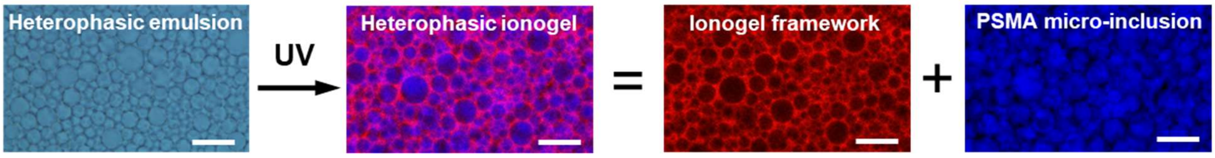

Supplementary Figure 1. Optical image of the heterophasic emulsion and confocal laser scanning microscopy image of the heterophasic ionogels with red-stained ionogel framework and blue-stained PSMA micro-inclusions. Scale bar, 10 μm.

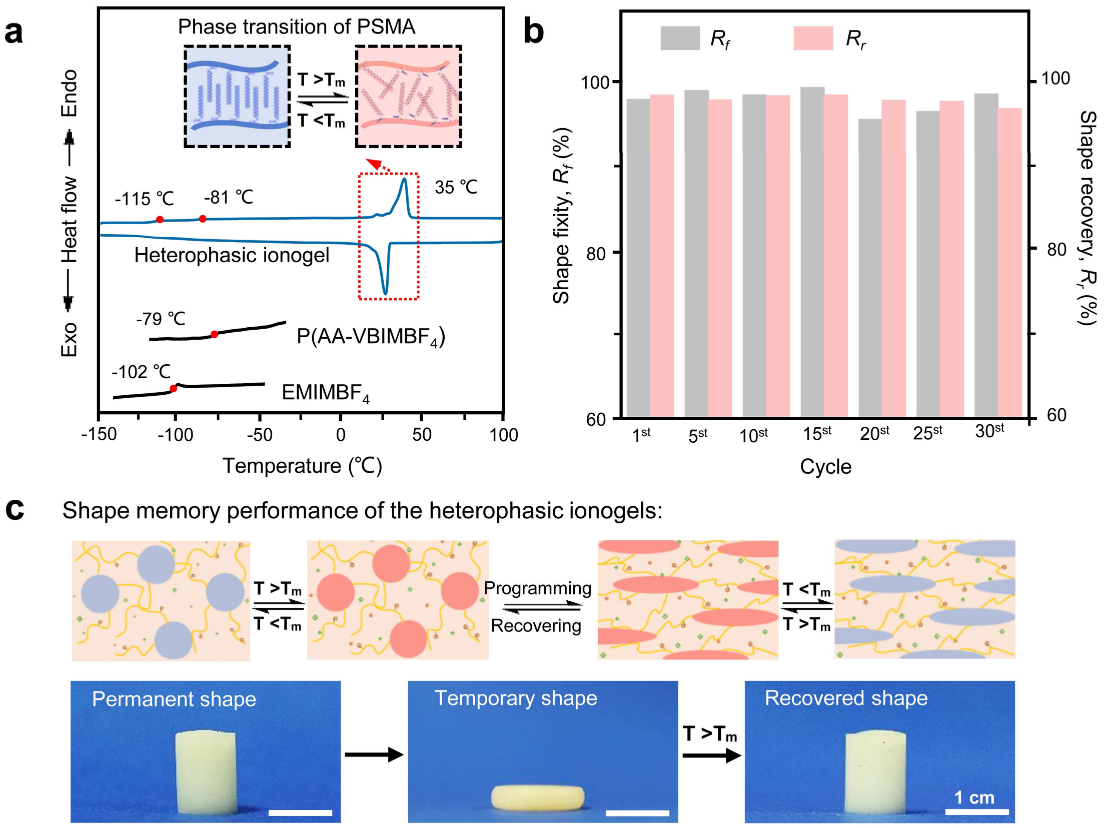

Supplementary Figure 2. Shape memory property of the heterophasic ionogels. (**a**) Differential scanning calorimetry (DSC) curves of the heterophasic ionogel and components: P(AA-VBIMBF$_4$) and EMIMBF$_4$. Illustration shows the phase transition of PSMA micro-inclusions. (**b**) Shape fixity (R_f) and shape recovery (R_r) of the heterophasic ionogels at different shape memory cycles. (**c**) shape memory performance of the heterophasic ionogels, scale bar, 1 cm.

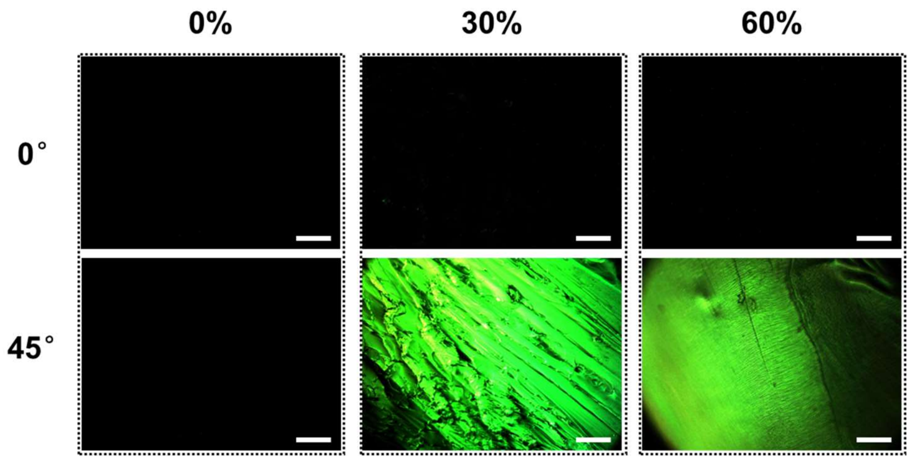

Supplementary Figure 3. POM images of the ionogel framework at different compression ratio, scale bar, 200 μm.

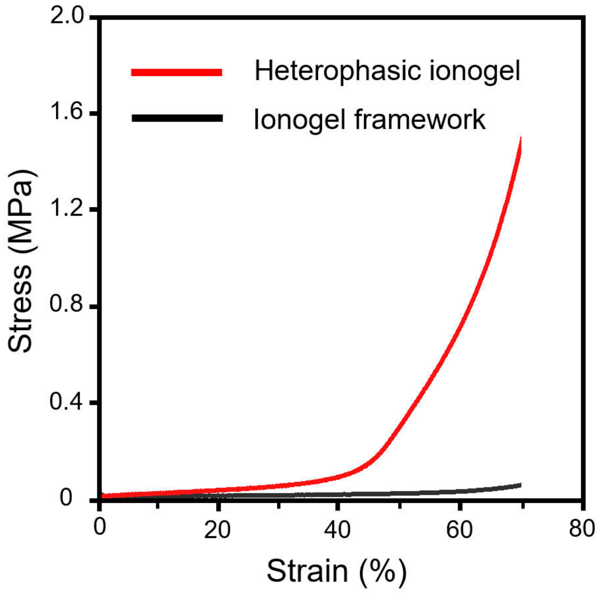

Supplementary Figure 4. Strain-stress curves of the original heterophasic ionogel and ionogel framework.

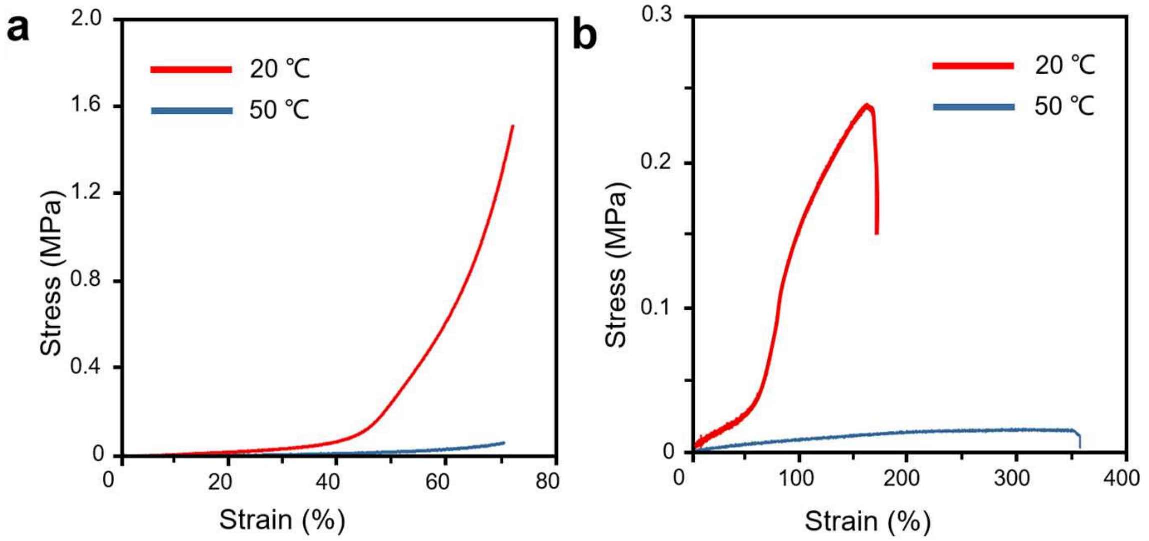

Supplementary Figure 5. Strain-stress curves of the original heterophasic ionogels under (**a**) compression and (**b**) stretching at different temperatures.

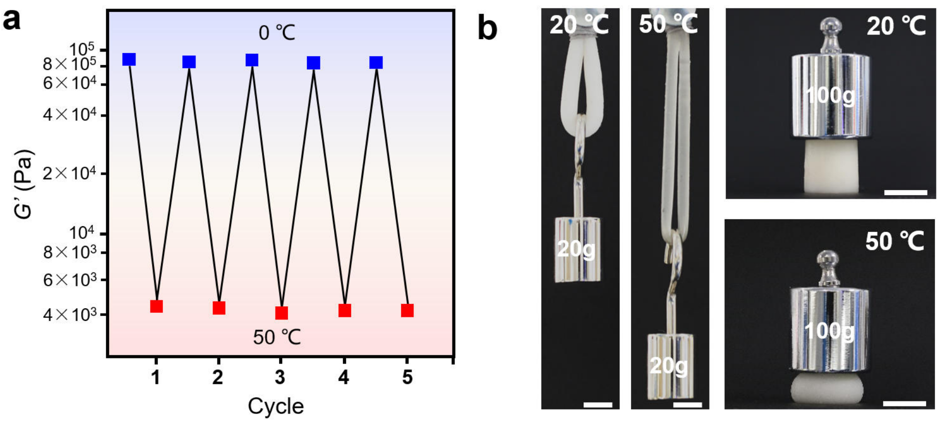

Supplementary Figure 6. Switchable mechanics of the original heterophasic ionogels. (**a**) Reversibly switched modulus of the heterophasic ionogel at 0 °C and 50 °C. (**b**) Pictures showing the heterophasic ionogels with low stretching/compression ratio at 20 °C and high at 50 °C. Scale bar, 1 cm.

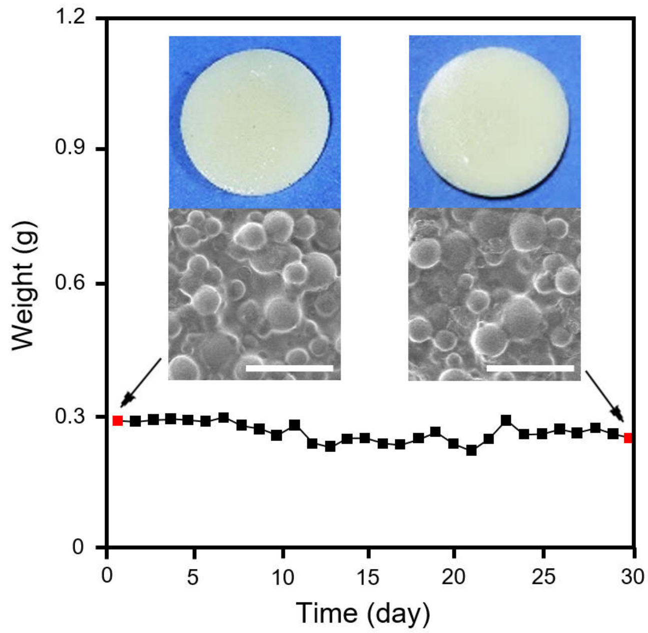

Supplementary Figure 7. Weight and structure changes during exposure in the air for 30 d revealing good stability of the original heterophasic ionogels. Scale bar, 10μm.

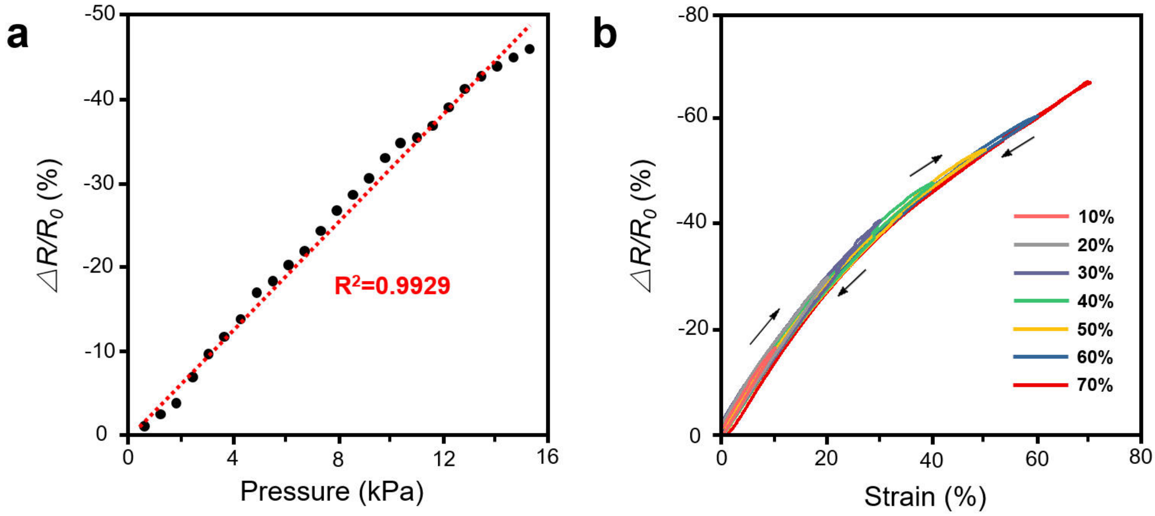

Supplementary Figure 8. Resistance change of the original ionogel sensors: (**a**) in pressure range of 0-16 kPa, (**b**) during compressing/releasing cycles.

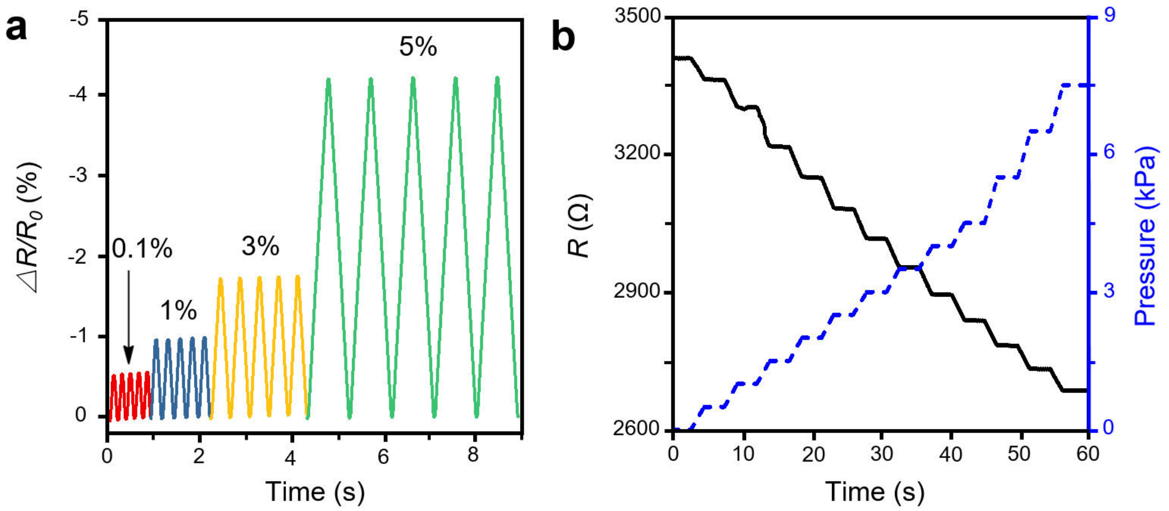

Supplementary Figure 9. Resistance changes of the original ionogel-based sensors under different strain (**a**) and pressure (**b**).

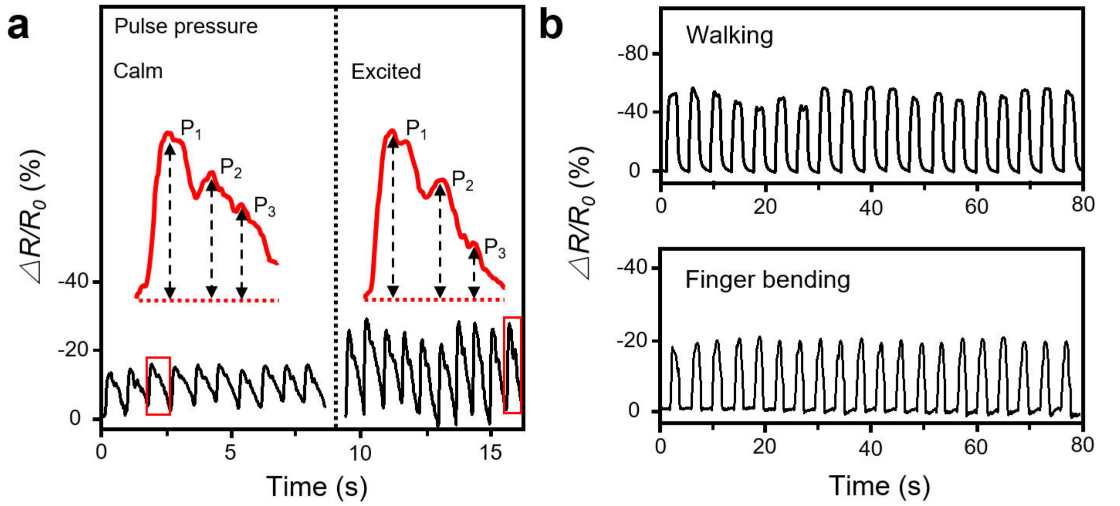

Supplementary Figure 10. Sensing ability of the original ionogel-based sensors. (**a**) Real-time pulse signals monitored by the ionogel sensor. The expanded pulse wave contains three peaks. (**b**) Response of the ionogel sensors in monitoring human motion (walking and finger bending).

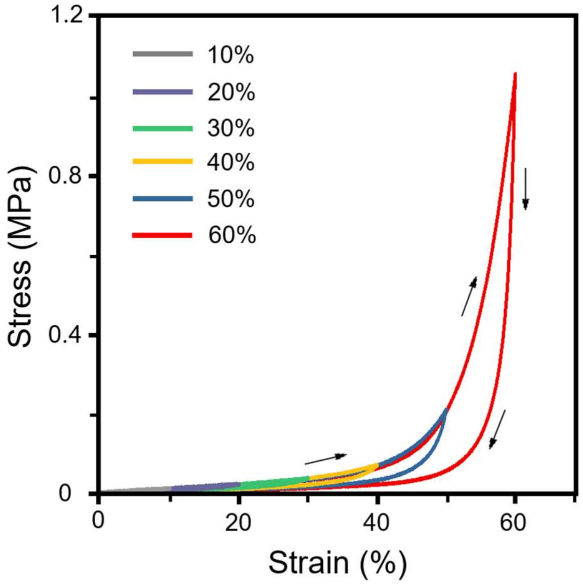

Supplementary Figure 11. The cyclic loading-unloading compressive tests of the original heterophasic ionogels with different compressive strains at 20 °C.

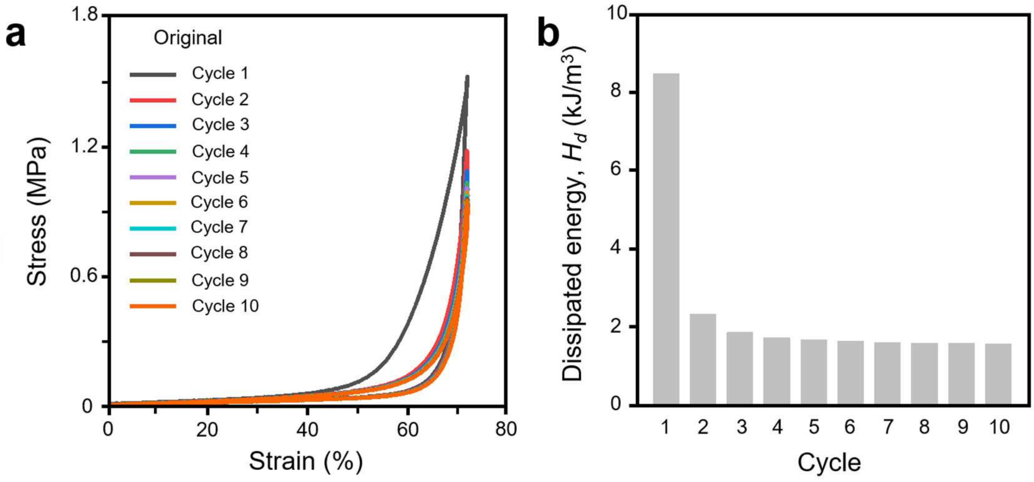

Supplementary Figure 12. The cyclic loading-unloading compressive tests of original ionogels. (**a**) Strain-stress curves during the cycle 1-10; (**b**) Dissipated energy of the original heterophasic ionogels at different loading-unloading cycles.

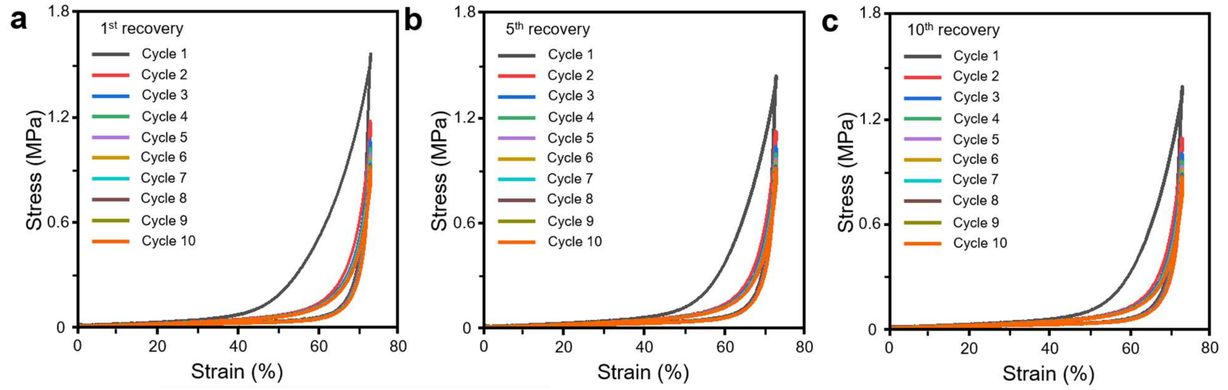

Supplementary Figure 13. The cyclic loading-unloading compressive tests of recovered ionogels: (**a**) 1st recovery; (**b**) 5th recovery and (**c**) 10th recovery.

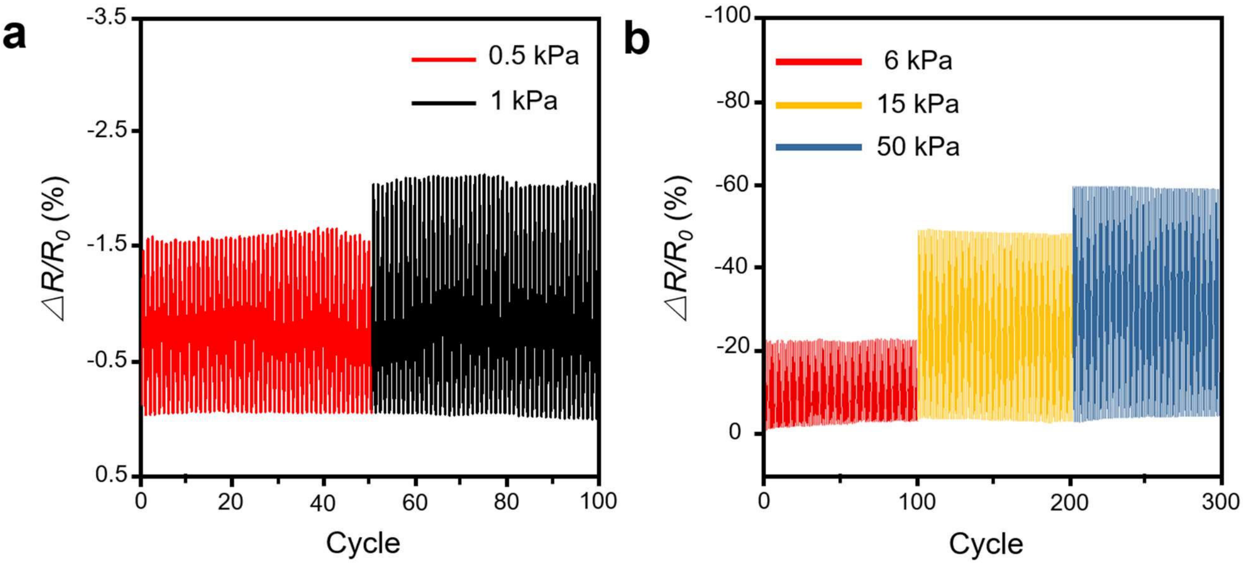

Supplementary Figure 14. The durable strain-sensitive test at (**a**) low pressure and (**b**) high pressure.

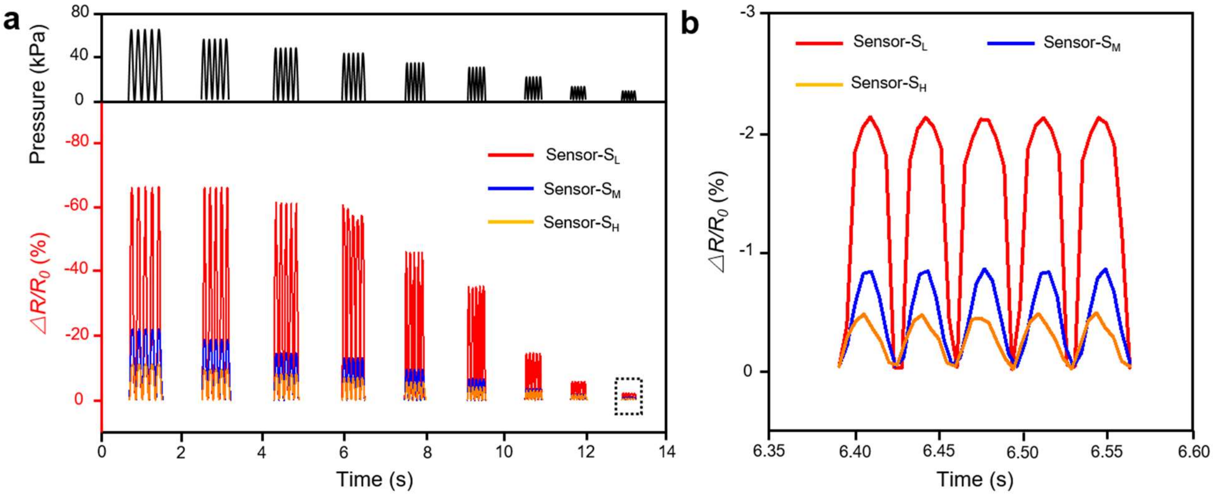

Supplementary Figure 15. Resistance response of the ionogel sensors under various pressure.
